# An Assessment of the Feasibility, Patient Acceptance, and Performance of Point-of-Care Transient Elastography for Metabolic-Dysfunction-Associated Steatotic Liver Disease (MASLD): A Systematic Review and Meta-Analysis

**DOI:** 10.3390/diagnostics14222478

**Published:** 2024-11-06

**Authors:** Taranika Sarkar Das, Xucong Meng, Mohamed Abdallah, Mohammad Bilal, Raiya Sarwar, Aasma Shaukat

**Affiliations:** 1 Department of Gastroenterology and Hepatology, New York University, New York, NY 10012, USA; xucong.meng@nyulangone.org (X.M.);; 2 Department of Gastroenterology and Hepatology, University of Minnesota, Minneapolis, MN 55455, USA

**Keywords:** point of care, MASLD, MASH, liver cirrhosis, liver transplant, Fibroscan

## Abstract

**Background**: Vibration-Controlled Transient Elastography (VCTE) with FibroScan is a non-invasive, reliable diagnostic tool for Metabolic-Dysfunction-Associated Steatotic Liver Disease (MASLD), enabling early detection and management to prevent severe liver diseases. VCTE’s ease and portability suit primary care, streamlining referrals, promoting lifestyle changes, reducing costs, and benefiting underserved communities. **Methods**: Studies on point-of-care VCTE were systematically reviewed, followed by meta-analysis using a random-effects model. Pooled proportions with 95% confidence intervals were reported, and heterogeneity was assessed using I2%. **Results**: A total of twenty studies from 14 countries, including 6159 patients, were analyzed, with three studies from France, two from the U.S., and four from China. The population had a slight male preponderance, with a mean age range of 35–73 years and a BMI range of 24.4–41.1%. The diagnostic accuracy for detecting any fibrosis (≥F1) was reported in four studies (*n* = 210) with an AUC of 0.74, sensitivity of 69.5%, and specificity of 70.6%. For significant fibrosis (≥F2), eight studies (*n* = 650) reported an AUC of 0.69, sensitivity of 81.7%, and specificity of 64.6%. Advanced fibrosis (≥F3) was evaluated in 10 studies (*n* = 619), with an AUC of 0.84, sensitivity of 88.1%, and specificity of 63.8%. Cirrhosis (F4) was assessed in nine studies (*n* = 533), with an AUC of 0.65, sensitivity of 87.5%, and specificity of 62.6%. Steatosis diagnoses across stages S1 to S3 showed increasing diagnostic accuracies, with AUCs of 0.85, 0.76, and 0.80, respectively. Probe type and BMI were significant covariates influencing diagnostic performance for both fibrosis and steatosis, while the percentage of male participants also showed significant associations. **Conclusions:** VCTE shows high diagnostic accuracy for fibrosis and steatosis in MASLD patients at the point of care. Future research should assess its implementation in fibroscan settings.

## 1. Introduction

Point-of-care tests are well established, offering benefits like real-time decision-making, cost-effectiveness, shorter hospital stays, and better patient satisfaction [1]. While liver biopsy remains the gold standard for diagnosing liver fibrosis, it has drawbacks such as invasiveness, sampling errors, and observer variability, especially at lower fibrosis stages [2]. This has led to growing interest in non-invasive diagnostic tools like Vibration-Controlled Transient Elastography (VCTE), performed by FibroScan, which is precise, quick, and suitable for point-of-care use [3]. Despite VCTE’s validation, the accuracy, yield, and adherence in point-of-care settings for Metabolic-Dysfunction-Associated Steatotic Liver Disease (MASLD) remain underexplored [4].

Although meta-analyses have evaluated VCTE’s accuracy in diagnosing nonalcoholic fatty liver disease (NAFLD), now reclassified as MASLD, research on its use in point-of-care settings is limited [5]. MASLD is a growing global health issue, with guidelines recommending initial blood test screenings followed by VCTE for those at risk [6]. However, the need for multiple appointments and referrals can hinder compliance [7]. While VCTE’s role in predicting liver-related events is increasingly studied, there is a lack of comparative research on its effectiveness in point-of-care settings versus traditional referrals [8]. This systematic review aims to fill that gap by evaluating point-of-care VCTE for MASLD and metabolic dysfunction-associated steatohepatitis (MASH), offering a comprehensive analysis of its diagnostic metrics.

## 2. Materials and Methods

### 2.1. Literature Search

A comprehensive literature search was conducted in Pubmed, EMBASE, and Cochrane Library with the assistance of a librarian in January 2023. The results were limited to the English language only. The following terms were searched: “Point of care tests gastroenterology, bedside tests in gastroenterology, liver stiffness measurement (LSM), transient elastography, VCTE, Fibro Scan, MASLD, MASH, fibrosis, and cirrhosis”. Additional studies were identified via a manual search for referenced studies and review articles. EndNote 20 software was used to manage the references. Duplicates were removed. We followed the Preferred Reporting Items for Systematic Reviews and Meta-Analysis (PRISMA) guidelines.

From the initial identification of 1302 studies in the databases, 19 and 214 studies were excluded, leaving 869 for screening. Of these, 823 were further removed for not meeting the inclusion criteria or being off-topic, resulting in 46 studies remaining. After a detailed review, 31 studies were excluded for not meeting the criteria or meeting the exclusion criteria, leaving 15 studies. Additionally, 31 hand-searched articles were included, totaling 49 studies. However, 17 of these were excluded for not being point-of-care studies, leaving 32 studies for analysis.

### 2.2. Selection Criteria

Studies were included based on the following criteria: (1) evaluation of the performance of transient elastography in predicting fibrosis, steatosis, and/or cirrhosis in MASLD/MASH at the point of care, published in peer-reviewed journals; (2) the use of liver biopsy as the gold standard for diagnosing liver fibrosis; (3) a focus on adult populations (≥18 years); (4) reporting of estimates for sensitivity, specificity, positive predictive value (PPV), negative predictive value (NPV), or receiver operating characteristic (ROC) curves for diagnosing fibrosis stages and differentiating MASH from simple steatosis.

### 2.3. Exclusion Criteria

Studies were excluded if they (i) were not point-of-care tests, (ii) addressed a different context of use, (iii) used an alternative index test as the gold standard, (iv) had insufficient data to calculate the diagnostic accuracy estimates, (v) included pediatric patients, (vi) were not published in English, or (vii) involved patients with coexisting liver diseases (e.g., MASLD and viral hepatitis in the same patient) or other causes of liver disease.

### 2.4. Data Extraction and Quality Assessment

Two reviewers (TSD and AS) independently evaluated a study’s eligibility, graded the study’s quality, and extracted data from the study. Any disagreements between the reviewers were resolved with detailed discussions between them, along with a third reviewer (RS). The parameters of our literature search included the author, year of publication, country, type of study region, patient gender, age, body mass index (BMI), number of patients, ultrasound-based transient elastography, % of diabetes, % of hypertension, % hyperlipidemia, prevalence of the fibrosis stage (as well as cutoff values to identify the fibrosis and steatosis stage), details of index text, performance indices of index test (cutoff values, sensitivity, specificity, PPV, NPV, AUROC), and histological fibrosis stages. The necessary data to calculate the number of true positives, false positives, true negatives, and false negatives were extracted. The quality of the included studies was independently appraised by two reviewers (TSD and AS) using the quality assessment of diagnostic accuracy studies (QUADAS) questionnaire. This could estimate the internal and external validity of diagnostic accuracy studies used in systematic reviews (Table 1).

### 2.5. Target Conditions

Liver fibrosis, steatosis, and MASH were the target conditions. Liver fibrosis was defined according to the MASH Clinical Research Network’s (CRN’s) histological classification. The diagnostic accuracy of VCTE was assessed in the following dichotomized groups: F0 vs. F1–4, F0–1 vs. F2–4, F0–2 vs. F3–4, F0–3 vs. F4, and MASH vs. simple steatosis. For this review, any definition of MASH was accepted. The following index tests were assessed in this review: VCTE (FibroScan^®^, Echosens, Paris, France) and its application as a point-of-care test.

### 2.6. Quality of Evidence Assessment

**Figure 1:**
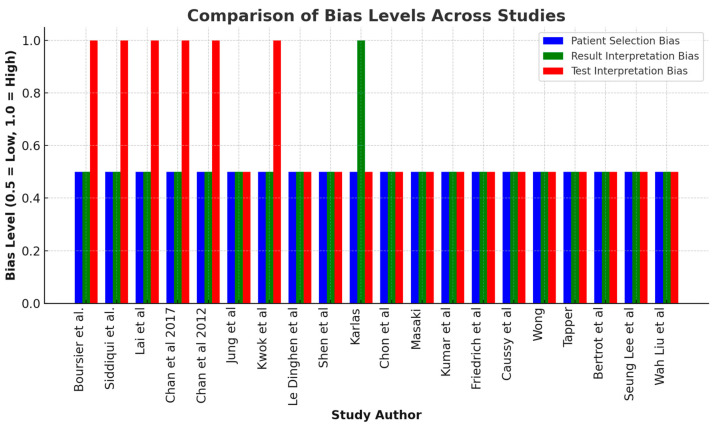
The bar chart above visualizes the comparison of bias levels across different studies. Blue bars represent the risk of bias in patient selection, which is consistently low across all studies. Green bars indicate the risk of bias in result interpretation, with only Karlas et al. [18] showing a high level of bias in this category. Red bars represent the risk of bias in test and reference test interpretation. Several studies, including Boursier et al. [9], Siddiqui et al. [10], Lai et al. [11], and both Chan et al. [12,13] studies, exhibit high bias here, while others, like Jung et al. [14] and Wong et al. [24], show low bias [15,17,18,19,20,21,22,23,25,27,28,29].

Table 1 and Figure 1 summarize the risk of bias in the studies. Most studies reported a low risk of patient selection bias, indicating representative samples with consecutive enrollment and enhancing their generalizability. However, some studies, like those by Karlas et al. [18], Boursier et al. [9], and Chan et al. [12,13], showed a high bias in VCTE result interpretation compared to liver biopsy, potentially affecting their accuracy. Additionally, studies such as Boursier et al. [9] and Lai et al. [11] introduced bias by not performing liver biopsies on all patients. Fortunately, no studies showed exclusion bias, ensuring comprehensive patient inclusion.

### 2.7. Evaluation of Diagnostic Accuracy

Classification tables were extracted and reconstructed to assess the diagnostic performance of the index test for each predefined target condition. For binary classifications, study-specific estimates of sensitivity, specificity, positive predictive value (PPV), negative predictive value (NPV), and both positive and negative likelihood ratios, along with their corresponding 95% confidence intervals, were computed. Forest plots were employed for a visual representation of sensitivity for each dichotomized group. Bivariate logit-normal random-effects models were used to estimate the average sensitivity, average specificity, and their corresponding variances and covariance. Summary receiver operating characteristic (sROC) curves were also generated. The I2 statistic was used to determine the heterogeneity between the studies, with significant heterogeneity defined with a value above 50% or *p*-value < 0.1.

### 2.8. Data Analysis

Covariate meta-regressions were performed to explore potential sources of heterogeneity. The degree of fibrosis, degree of steatosis, and probe type were evaluated as potential covariates for each dichotomized group. Additionally, the mean BMI and percentage of male participants were assessed as potential covariates for each study. Reitsma models were constructed, both with and without the inclusion of these covariates for each fibrosis stage group, and were compared using the likelihood ratio test statistic. All analyses were conducted using the statistical software R (Version 4.3.1; R Foundation for Statistical Computing, Vienna, Austria).

## 3. Results

### 3.1. Study Characteristics

Twenty studies from 14 different countries were included in the analysis. Three studies were from France, two from the US, and four from China. One was a cross-sectional study, while seventeen were cohort studies (Table 2).

### 3.2. Patient Characteristics

In total, 6369 patients were included in the analysis (Table 2). There was a slight preponderance of males, a mean age range 35–61 years, and a BMI range of 24.4–41.1%.

### 3.3. Diagnosis of Any Fibrosis (F0 vs. F1–4)

**Figure 2:**
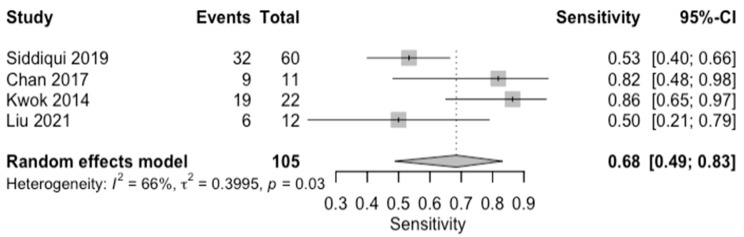
Forest plots for sensitivity of any degree of fibrosis (F0 vs. F1–4) along with their 95% confidence intervals (CIs). The plot includes four studies: Siddiqui 2019 [9], Chan 2017 [13], Kwok 2015 [15], and Liu 2021 [28]. Each study’s sensitivity is represented by a square, with the square size reflecting the study weight in the random effects model. Horizontal lines indicate the 95% CI for each study. The diamond at the bottom represents the pooled sensitivity estimate and its 95% CI, based on the random effects model. Heterogeneity among studies is quantified by I^2^ (66%) and τ^2^ (0.3995), with a *p*-value of 0.03 indicating significant heterogeneity.

**Figure 3:**
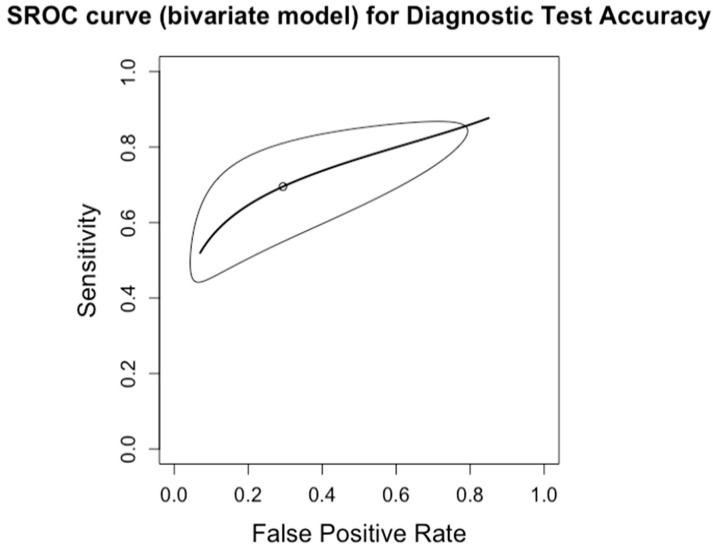
Summary Receiver Operating Characteristic (SROC) curve for diagnostic test accuracy of any degree of fibrosis (F0 vs. F1–4). The curve plots sensitivity (y-axis) against false positive rate (x-axis), providing an overall measure of test performance across studies. The central curve represents the relationship between sensitivity and false positive rate, while the surrounding shaded region illustrates the 95% confidence region, indicating the variability in diagnostic accuracy.

The diagnostic accuracy in detecting any degree of fibrosis (≥F1) was investigated by four studies (*n* = 210) (Table 3; Figure 2 for forest plot). The respective AUCs, sensitivities, specificities, positive likelihood ratios, negative likelihood ratios, and diagnostic odds ratios for a diagnosing stage ≥ F1 were as follows: 0.74, 69.5%, 70.6%, 2.93, 0.47, and 6.62. The summary point estimate of the mean is shown in Figure 3.

### 3.4. Diagnosis of Significant Fibrosis (F0–1 vs. F2–4)

**Figure 4:**
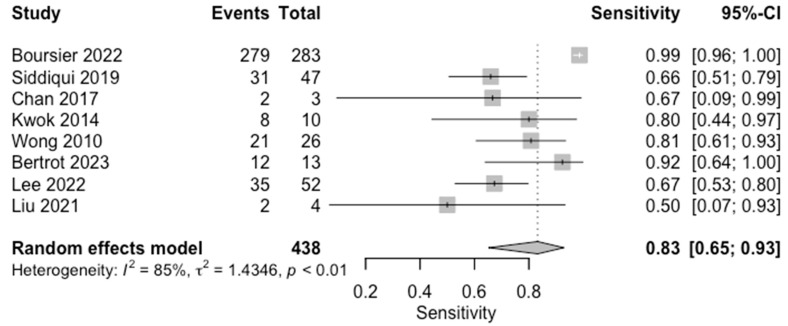
Forest plots for sensitivity of significant fibrosis (F0–1 vs. F2–4) along with their 95% confidence intervals (CIs). The plot includes four studies: Boursier 2022 [9], Siddiqui 2019 [10], Chan 2017 [13], Kwok 2014 [15], Wong 2010 [24], Bertrot 2023 [26], Lee 2022 [27], and Liu 2021 [28]. Each study’s sensitivity is represented by a square, with the square size reflecting the study weight in the random effects model. Horizontal lines indicate the 95% CI for each study. The diamond at the bottom represents the pooled sensitivity estimate and its 95% CI, based on the random effects model. Heterogeneity among studies is quantified by I^2^ (85%) and τ^2^ (1.4346), with a *p*-value of <0.01 indicating significant heterogeneity.

**Figure 5:**
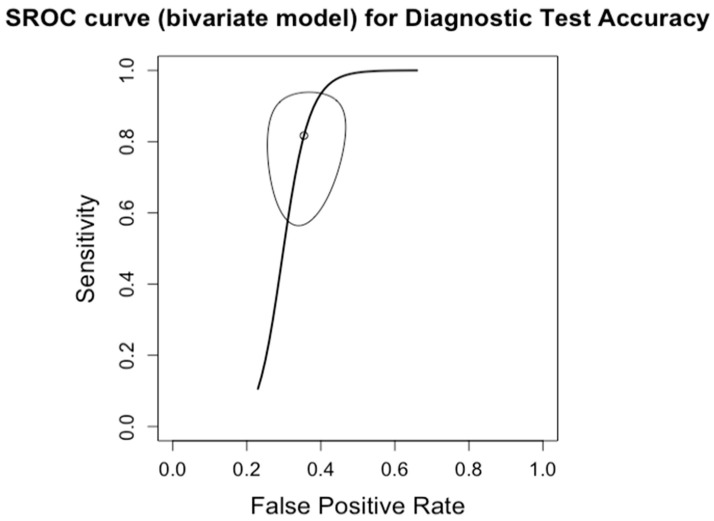
Summary Receiver Operating Characteristic (SROC) curve for diagnostic test accuracy of fignificant fibrosis (F0–1 vs. F2–4). The curve plots sensitivity (y-axis) against false positive rate (x-axis), providing an overall measure of test performance across studies. The central curve represents the relationship between sensitivity and false positive rate, while the surrounding shaded region illustrates the 95% confidence region, indicating the variability in diagnostic accuracy.

The diagnostic accuracy in detecting significant fibrosis (≥F2) was investigated by eight studies (*n* = 650) (Table 3; Figure 4 for forest plot). The respective AUCs, sensitivities, specificities, positive likelihood ratios, negative likelihood ratios, and diagnostic odds ratios for a diagnosing stage ≥ F2 were as follows: 0.69, 81.7%, 64.6%, 2.30, 0.30, and 9.28. The summary point estimate of the mean is shown in Figure 5.

### 3.5. Diagnosis of Advanced Fibrosis (F0–2 vs. F3–4)

**Figure 6:**
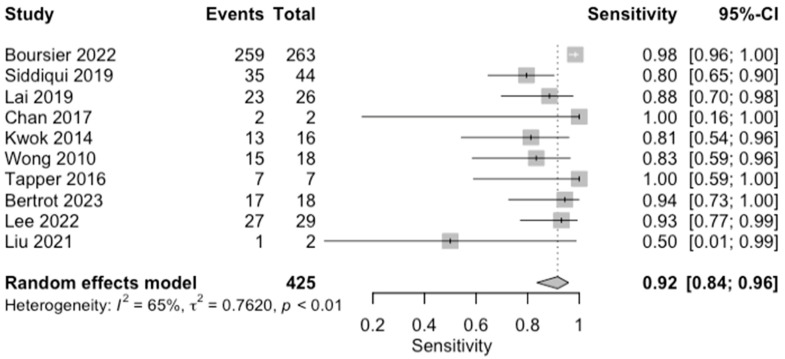
Forest plots for sensitivity of advanced fibrosis (F0–2 vs. F3–4) along with their 95% confidence intervals (CIs). The plot includes four studies: Boursier 2022 [9], Siddiqui 2019 [10], Lai 2019 [11], Chan 2017 [13], Kwok 2014 [15], Wong 2010 [24], Tapper 2016 [25], Bertrot 2023 [26], Lee 2022 [27], and Liu 2021 [28]. Each study’s sensitivity is represented by a square, with the square size reflecting the study weight in the random effects model. Horizontal lines indicate the 95% CI for each study. The diamond at the bottom represents the pooled sensitivity estimate and its 95% CI, based on the random effects model. Heterogeneity among studies is quantified by I^2^ (65%) and τ^2^ (0.7620), with a *p*-value of <0.01 indicating significant heterogeneity.

**Figure 7:**
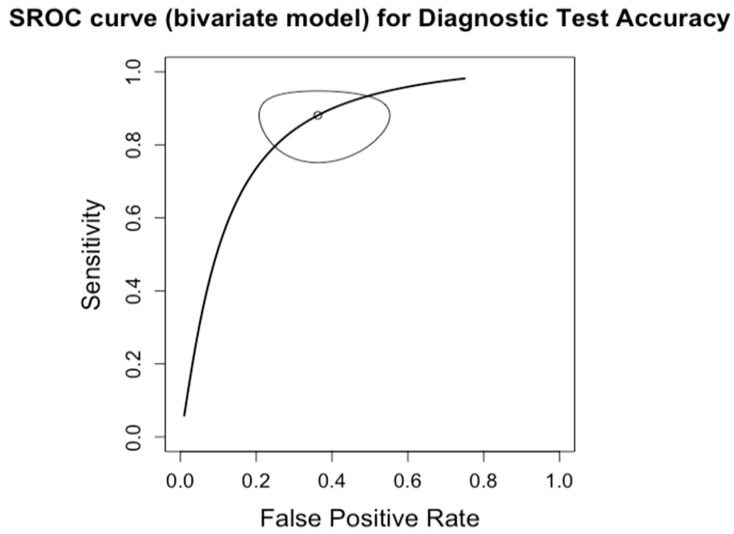
Summary Receiver Operating Characteristic (SROC) curve for diagnostic test accuracy of advanced fibrosis (F0–2 vs. F3–4). The curve plots sensitivity (y-axis) against false positive rate (x-axis), providing an overall measure of test performance across studies. The central curve represents the relationship between sensitivity and false positive rate, while the surrounding shaded region illustrates the 95% confidence region, indicating the variability in diagnostic accuracy.

The diagnostic accuracy in detecting advanced fibrosis (≥F3) was investigated by ten studies (*n* = 619) (Table 3; Figure 6 for forest plot). The respective AUCs, sensitivities, specificities, positive likelihood ratios, negative likelihood ratios, and diagnostic odds ratios for a diagnosing stage ≥ F3 were as follows: 0.84, 88.1%, 63.8%, 2.50, 0.20, and 14.60. The summary point estimate of the mean is shown in Figure 7.

### 3.6. Diagnosis of Cirrhosis (F0–3 vs. F4)

**Figure 8:**
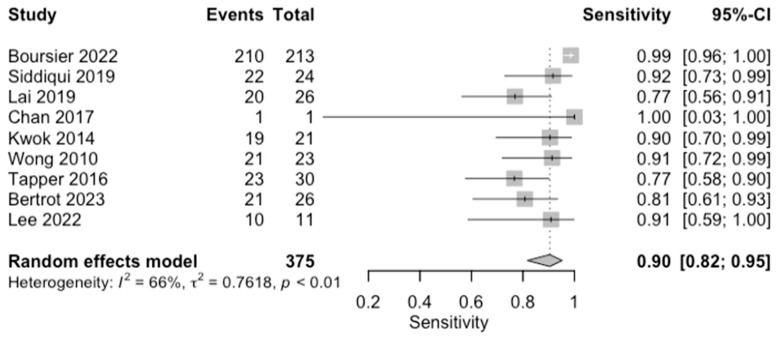
Forest plots for sensitivity of cirrhosis (F0–3 vs. F4) along with their 95% confidence intervals (CIs). The plot includes four studies: Boursier 2022 [9], Siddiqui 2019 [10], Lai 2019 [11], Chan 2017 [13], Kwok 2014 [15], Wong 2010 [24], Tapper 2016 [25], Bertrot 2023 [26], Lee 2022 [27], and Liu 2021 [28]. Each study’s sensitivity is represented by a square, with the square size reflecting the study weight in the random effects model. Horizontal lines indicate the 95% CI for each study. The diamond at the bottom represents the pooled sensitivity estimate and its 95% CI, based on the random effects model. Heterogeneity among studies is quantified by I^2^ (65%) and τ^2^ (0.7620), with a *p*-value of <0.01 indicating significant heterogeneity.

**Figure 9:**
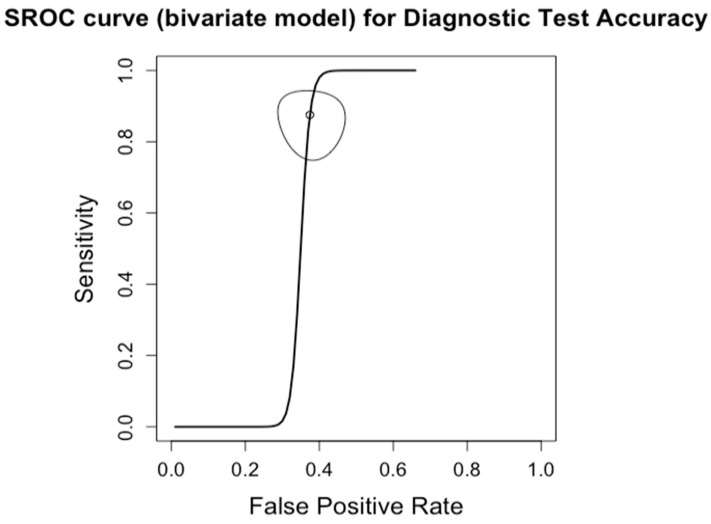
Summary Receiver Operating Characteristic (SROC) curve for diagnostic test accuracy of cirrhosis (F0–3 vs. F4). The curve plots sensitivity (y-axis) against false positive rate (x-axis), providing an overall measure of test performance across studies. The central curve represents the relationship between sensitivity and false positive rate, while the surrounding shaded region illustrates the 95% confidence region, indicating the variability in diagnostic accuracy.

The diagnostic accuracy in detecting cirrhosis (=F4) was investigated by nine studies (*n* = 543) (Table 3; Figure 8 for forest plot). The respective AUCs, sensitivities, specificities, positive likelihood ratios, negative likelihood ratios, and diagnostic odds ratios for diagnosing stage = F4 were as follows: 0.65, 87.5%, 62.6%, 2.34, 0.21, and 12.70. The summary point estimate of the mean is shown in Figure 9.

### 3.7. Diagnosis of Mild Steatosis (CAP < 33%, S1)

**Figure 10:**
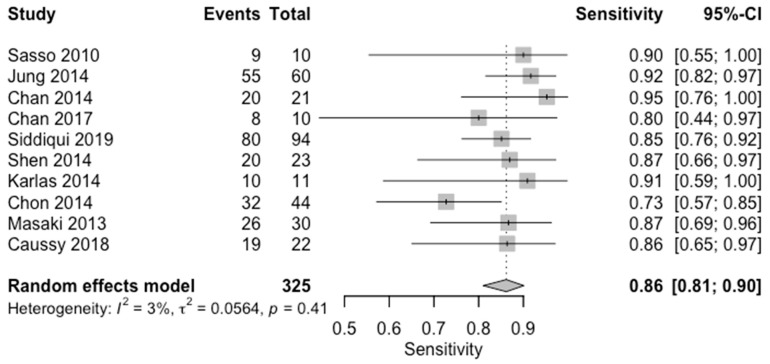
Forest plots for sensitivity of mild steatosis (CAP < 33%, S1) along with their 95% confidence intervals (CIs). The plot includes four studies: Sasso 2010 [16], Jung 2014 [14], Chan 2014 [12], Chan 2017 [13], Siddiqui 2019 [10], Shen 2014 [17], Karlas 2014 [18], Chon 2014 [19], Masaki 2013 [20], and Caussy 2018 [23]. Each study’s sensitivity is represented by a square, with the square size reflecting the study weight in the random effects model. Horizontal lines indicate the 95% CI for each study. The diamond at the bottom represents the pooled sensitivity estimate and its 95% CI, based on the random effects model. Heterogeneity among studies is quantified by I^2^ (3%) and τ^2^ (0.0564), with a *p*-value of 0.41 indicating low heterogeneity.

**Figure 11:**
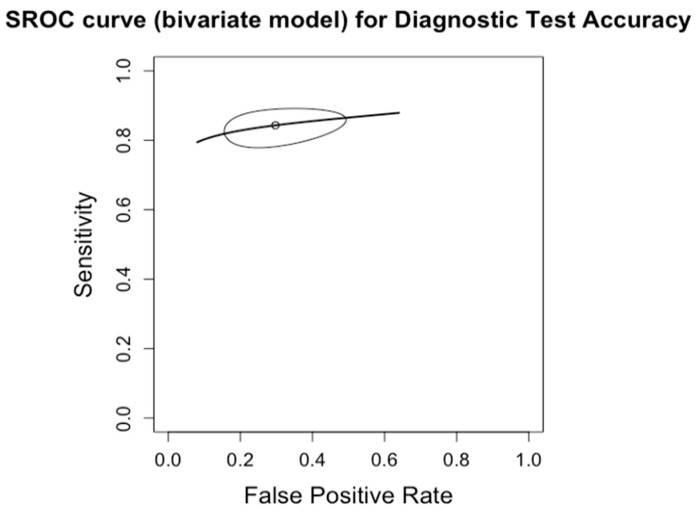
Summary Receiver Operating Characteristic (SROC) curve for diagnostic test accuracy of mild steatosis (CAP < 33%, S1). The curve plots sensitivity (y-axis) against false positive rate (x-axis), providing an overall measure of test performance across studies. The central curve represents the relationship between sensitivity and false positive rate, while the surrounding shaded region illustrates the 95% confidence region, indicating the variability in diagnostic accuracy.

The diagnostic accuracy in detecting mild steatosis (S1) was investigated by ten studies (*n* = 510) (Table 3; Figure 10 for forest plot). The respective AUCs, sensitivities, specificities, positive likelihood ratios, negative likelihood ratios, and diagnostic odds ratios for diagnosing stage S1 were as follows: 0.85, 84.3%, 70.3%, 2.94, 0.23, and 13.40. The summary point estimate of the mean is shown in Figure 11.

### 3.8. Diagnosis of Moderate Steatosis (CAP 34–66%, S2)

**Figure 12:**
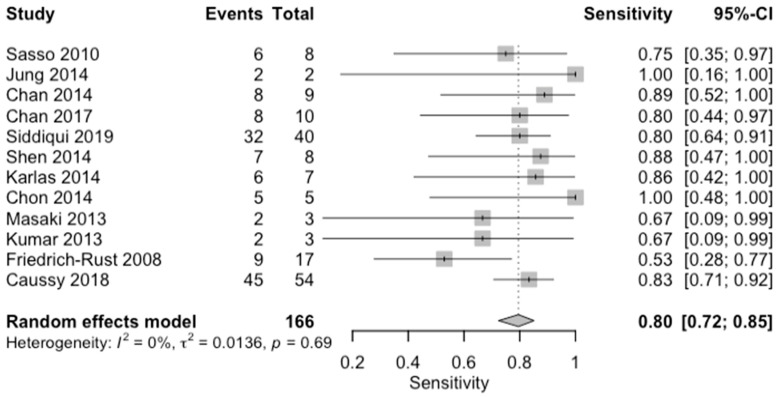
Forest plots for sensitivity of moderate steatosis (CAP 34–66%, S2) along with their 95% confidence intervals (CIs). The plot includes four studies: Sasso 2010 [16], Jung 2014 [14], Chan 2014 [12], Chan 2017 [13], Siddiqui 2019 [10], Shen 2014 [17], Karlas 2014 [18], Chon 2014 [19], Masaki 2013 [20], Kumar 2013 [21], Friedrich-Rust 2008 [22], and Caussy 2018 [23]. Each study’s sensitivity is represented by a square, with the square size reflecting the study weight in the random effects model. Horizontal lines indicate the 95% CI for each study. The diamond at the bottom represents the pooled sensitivity estimate and its 95% CI, based on the random effects model. Heterogeneity among studies is quantified by I^2^ (0%) and τ^2^ (0.0136), with a *p*-value of 0.69 indicating low heterogeneity.

**Figure 13:**
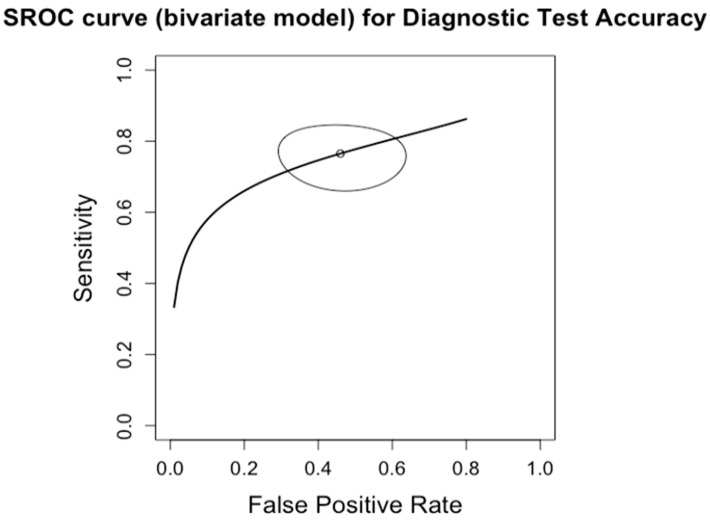
Summary Receiver Operating Characteristic (SROC) curve for diagnostic test accuracy of moderate steatosis (CAP 34–66%, S2). The curve plots sensitivity (y-axis) against false positive rate (x-axis), providing an overall measure of test performance across studies. The central curve represents the relationship between sensitivity and false positive rate, while the surrounding shaded region illustrates the 95% confidence region, indicating the variability in diagnostic accuracy.

The diagnostic accuracy in detecting moderate steatosis (S2) was investigated by thirteen studies (*n* = 309) (Table 3; Figure 12 for forest plot). The respective AUCs, sensitivities, specificities, positive likelihood ratios, negative likelihood ratios, and diagnostic odds ratios for diagnosing stage S2 were as follows: 0.75, 76.8%, 54.0%, 1.70, 0.45, and 4.11. The summary point estimate of the mean is shown in Figure 13.

### 3.9. Diagnosis of Severe Steatosis (CAP > 67%, S3)

**Figure 14:**
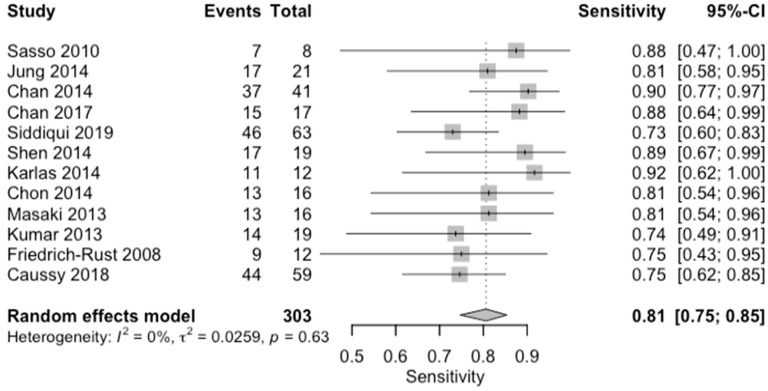
Forest plots for sensitivity of severe steatosis (CAP > 67%, S3) along with their 95% confidence intervals (CIs). The plot includes four studies: Sasso 2010 [16], Jung 2014 [14], Chan 2014 [12], Chan 2017 [13], Siddiqui 2019 [10], Shen 2014 [17], Karlas 2014 [18], Chon 2014 [19], Masaki 2013 [20], Kumar 2013 [21], Friedrich-Rust 2008 [22], and Caussy 2018 [23]. Each study’s sensitivity is represented by a square, with the square size reflecting the study weight in the random effects model. Horizontal lines indicate the 95% CI for each study. The diamond at the bottom represents the pooled sensitivity estimate and its 95% CI, based on the random effects model. Heterogeneity among studies is quantified by I^2^ (0%) and τ^2^ (0.0259), with a *p*-value of 0.63 indicating low heterogeneity.

**Figure 15:**
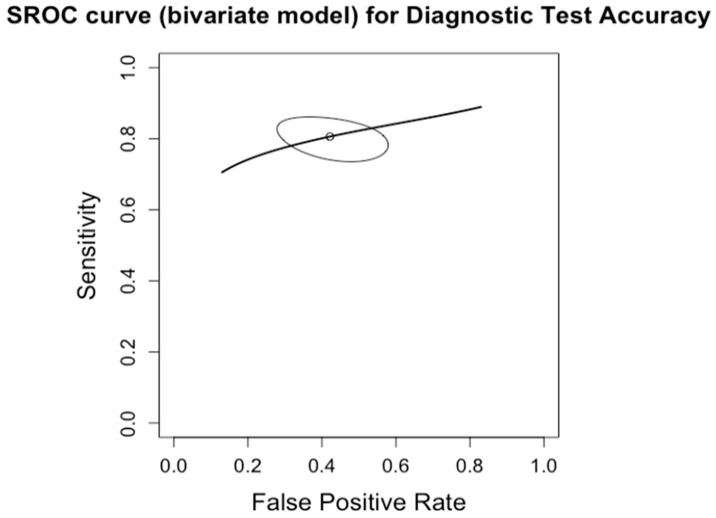
Summary Receiver Operating Characteristic (SROC) curve for diagnostic test accuracy of moderate steatosis (CAP >67%, S3). The curve plots sensitivity (y-axis) against false positive rate (x-axis), providing an overall measure of test performance across studies. The central curve represents the relationship between sensitivity and false positive rate, while the surrounding shaded region illustrates the 95% confidence region, indicating the variability in diagnostic accuracy.

The diagnostic accuracy in detecting severe steatosis (S3) was investigated by twelve studies (*n* = 518) (Table 3; Figure 14 for forest plot). The respective AUCs, sensitivities, specificities, positive likelihood ratios, negative likelihood ratios, and diagnostic odds ratios for diagnosing stage S3 were as follows: 0.80, 80.6%, 57.9%, 1.95, 0.35, and 6.07. The summary point estimate of the mean is shown in Figure 15.

The probe type was identified as a significant covariate influencing the diagnostic performance for both any fibrosis and significant fibrosis, as well as for mild and severe steatosis (Table 4). BMI was a significant covariate for steatosis but not for fibrosis (Table 5). The percentage of male participants demonstrated a significant association with both fibrosis and steatosis (Table 6). However, the degree of fibrosis and steatosis did not have a statistically significant impact on the diagnostic performance of VCTE (Table 7).

### 3.10. Adherence to POC VCTE

Table 2 summarizes the findings from six studies that evaluated the completion rate of Vibration-Controlled Transient Elastography (VCTE) in patients who were offered this option. The adherence rates were predominantly high, exceeding 90% in the majority of studies.

## 4. Discussion

Numerous meta-analyses have assessed the accuracy of Vibration-Controlled Transient Elastography (VCTE) in diagnosing NAFLD [30], but none have focused on its use in a point-of-care setting. MASLD, formerly known as NAFLD, is a rapidly growing global health issue. Current guidelines recommend using simple blood tests to screen at-risk populations for fibrosis, followed by referral for VCTE if elevated scores are detected [31]. However, challenges such as the need for multiple appointments and traveling to urban centers for VCTE or MRE may result in non-compliance. To date, no comparative study has examined VCTE performed at the point of care versus the traditional referral route.

VCTE is being increasingly explored for its potential to predict liver-related events (LREs) [32,33]. In this systematic review, we investigate the use of point-of-care VCTE for diagnosing MASLD/MASH. This synthesis represents the most comprehensive pooled analysis of diagnostic metrics for point-of-care VCTE in MASLD to date. Our findings indicate that point-of-care VCTE exhibits high diagnostic accuracy, with sensitivities ranging from 76% to 89% and specificities from 67% to 73% for F1 to F4, underscoring its value as a critical point-of-care test.

The AASLD (AASLD Practice Guidance on the Clinical Assessment and Management of Nonalcoholic Fatty Liver Disease) has recently updated its guidelines on high-risk populations [34].

Clinicians are advised to screen patients with MASLD for type 2 diabetes, particularly advanced fibrosis, due to the increased risk of nonalcoholic steatohepatitis (MASH). Screening for advanced fibrosis is also recommended in patients with obesity. The adherence rate for Vibration-Controlled Transient Elastography (VCTE) is notably high in comparison to other tests used for diagnosing fibrosis and steatosis. In a large multicenter prospective study that utilized Vibration-Controlled Transient Elastography (VCTE) for diagnosing nonalcoholic fatty liver disease (MASLD), there was a high compliance rate. Out of 1696 scans performed, only 11 patients did not show up for the procedure. This translates to a compliance rate of approximately 99%. This high acceptance rate indicates the feasibility and patient acceptance of VCTE in clinical settings for MASLD diagnosis [35]. According to research, VCTE’s compliance rate was found to be 93.4% (142 out of 152 cases), which is higher than the 85% compliance rate observed for MRI–Proton Density Fat Fraction (MRI-PDFF) in one study [36]. In another study, the compliance rate for MRI-PDFF was reported to be 85% (103 out of 120 cases) [37,38].

Kan et al. conducted a study to determine patient preferences between VCTE and liver biopsy (LB), along with their willingness to pay for VCTE services. In British Columbia, where liver biopsy is covered by public funding, the study found that VCTE was the more favored method for assessing liver fibrosis among patients. Furthermore, a majority of these patients were willing to cover the costs of VCTE themselves [39].

The current prices of various diagnostic tools for liver fibrosis are as follows: MRE scan costs USD 250.00, VCTE (FibroScan) is USD 140.33, and biopsy amounts to USD 1372.45.

Additionally, a study by Gomez et al. demonstrated that VCTE is more cost-effective than both MRE and liver biopsy over a five-year period [40].

Research indicates that the initial attendance for Vibration-Controlled Transient Elastography (VCTE) is high, but attendance tends to decrease with follow-up appointments. One study showed a follow-up response rate of 50.9% (59 out of 116) [41]. Tapper et al. reported a 91.4% (169 out of 185) adherence at 3 months, dropping to 53% (87 out of 164) at 6 months [25]. Wah Liu et al. observed adherence rates of 94.6% at 1 year, 100% at 2 years, 94.6% at 3 years, 91.9% at 4 years, and 64.9% at 5 years [28]. Another study reported a 6-mnth follow-up rate of 86% (43 out of 50). This trend highlights a decline in follow-up engagement over time [42].

Integrating point-of-care testing for Vibration-Controlled Transient Elastography (VCTE) during primary care physician/general gastroenterologist visits could minimize barriers such as the need for multiple appointments. This approach would streamline the completion of VCTE without compromising the test’s diagnostic accuracy. We found adherence rates to POC VCTE ranging from 54 to 98.3% but mostly above 90%.

This finding underscores the significance of VCTE (Vibration-Controlled Transient Elastography) as a valuable tool in the point-of-care setting for evaluating MASLD [43].

Several multivariate analyses have been initiated to enhance the sensitivity and predictive efficacy of simple laboratory tests for liver fibrosis. These include the FIB-4 index, the MASLD fibrosis score, the ELF (Enhanced Liver Fibrosis) test, the BARD score, the FibroTest, and the Hepascore. The ELF test, a commercially available algorithm, incorporates three serum biomarkers: hyaluronic acid (HA), the N-terminal pro-peptide of collagen type III (PIIINP), and tissue inhibitor of metalloproteinase-1 (TIMP1). A recent prospective study compared the ELF test with FibroTest and Elastography in 289 patients. While the results from the ELF test and FibroTest were not significantly different from those of liver stiffness measurements in intention-to-diagnose analyses (AUROC for transient elastography, 0.90), discrepancies were observed in the per-protocol analysis (AUROC for transient elastography, 0.97). The cutoff value of 10.5 for the ELF test was excellent for ruling out advanced fibrosis, with a negative predictive value (NPV) of 98%. However, it could not definitively confirm a diagnosis of advanced fibrosis due to a positive predictive value (PPV) of 60% [44,45]. Considering that VCTE is notably the most cost-effective among the non-invasive tests [46,47] and surpasses blood fibrosis tests in accuracy [48], point-of-care VCTE emerges as an economical option with the potential to address healthcare disparities by enabling the early diagnosis and treatment of MASLD within underserved, unrepresented, and marginalized communities. Its user and resource-friendly nature with easy portability allows for wider implementation in primary care settings and community settings, facilitating large-scale screening and monitoring. This enables primary care physicians and gastroenterologists alike to assess liver fibrosis at the point of care and allows for the early implementation of awareness and lifestyle modifications. It can streamline referrals by reducing multiple appointments, minimizing scheduling complexities and the risk of missing work multiple times. It can avoid unnecessary specialist consultations for mild fibrosis. Incorporating TE could lead to better early MASLD management and increased patient education and engagement. It can help in liver disease monitoring at the point of care to avoid MASH, cirrhosis, and hepatocellular carcinoma and help in healthcare cost savings. It is particularly advantageous for underserved populations, given its cost-effectiveness compared to alternatives such as MRI or liver biopsy. It is important to note that resistance toward MR elastography is often driven by insurance coverage or patient preferences. Additionally, this approach eliminates the requirement for additional travel, which can be a significant obstacle, especially when traveling to a city is not feasible. Yoneda et al. highlighted that referring patients to hepatology for elastographic examinations can decrease patient follow-up and attendance [48]. Thus, employing a point-of-care (POC) VCTE during clinic appointments could additionally diminish the necessity for recurring visits, minimize delays, and prevent loss of follow-up. Introducing visual reports during patients’ visits, VCTE can enhance their engagement in liver care by providing a pictorial representation of their liver condition. This can lead to improved follow-up appointments, better compliance with treatment plans, and increased overall involvement of patients in managing their liver health. It has been seen that combining TE with visual reports enhances MASLD patient satisfaction by improving clarity and understanding [49].

Our study has several limitations that warrant consideration. Several of the included studies were retrospective, observational, and population-based. It has been observed that CAP’s accuracy diminishes at higher BMIs, and although using an XL probe can partially mitigate this issue, it still remains less accurate compared to individuals with lower BMIs.

Moreover, CAP may not be sufficiently reliable for grading steatosis in patients with MASLD. Its diagnostic performance in identifying severe steatosis is sub-optimal, and its ability to differentiate between steatosis grade 2 and grade 3 was found to be unsatisfactory, similar to evidence by studies conducted by Sasso et al. and Ledinghen et al. [29,50]. Our research revealed that steatosis grade 3 or high CAP values serve as independent risk factors for discordant results between a liver biopsy and CAP. Another limitation arises from the lack of universally standardized cutoffs for MASLD. As a result, our study encountered a range of cutoff values being used, which could potentially impact the overall accuracy and consistency of the results. Also, there was variability among the included studies and technical differences that were not fully addressed, which could have influenced the heterogeneity observed in the results. Nevertheless, this study offers significant insights into the combined diagnostic accuracy of point-of-care VCTE for MASLD.

## 5. Conclusions

In conclusion, point-of-care Vibration-Controlled Transient Elastography (VCTE) emerges as a valuable tool for the non-invasive assessment of liver fibrosis in patients with Metabolic-Dysfunction-Associated Steatotic Liver Disease (MASLD) and Metabolism-Associated Steatohepatitis (MASH). This systematic review highlights the high diagnostic accuracy of VCTE, with sensitivity and specificity levels that make it a reliable option for staging liver fibrosis at the point of care. The integration of VCTE into routine clinical practice could significantly reduce the barriers associated with traditional referral-based approaches, such as the need for multiple appointments and the resulting non-compliance. Moreover, the high adherence rates observed in point-of-care settings underscore the feasibility and acceptance of VCTE among patients.

By streamlining the diagnostic process and enabling earlier intervention, point-of-care VCTE has the potential to improve patient outcomes, particularly in high-risk populations. As the global burden of MASLD continues to grow, the adoption of VCTE in primary care and community settings could play a crucial role in large-scale screening and monitoring efforts. Ultimately, the findings from this review support the broader implementation of point-of-care VCTE as a cost-effective, accessible, and efficient strategy for managing liver disease in diverse clinical environments.

## Figures and Tables

**Table 1:** Risk of Bias.

Study Author	Was a Consecutive or Random Sample of Patients Enrolled?	Was a Case–Control Design Avoided?	Did the Study Avoid Inappropriate Exclusions?	Were the Index Test Results (VCTE) Interpreted Without Knowledge of the Results of the Reference Standard (Liver Biopsy)?	If a Threshold Was Used, Was It Pre-Specified?	Did All Patients Receive a Reference Standard (Liver Biopsy)?	Were All Patients Included in the Analysis?	Risk of Bias for Patient Selection? High, Low, or Unclear	Risk of Bias for Result Interpretion? High, Low, or Uncertain	Risk of Bias for Interpretion of Test and Reference Test? High, Low, or Unclear
Boursier et al. [9]	consecutive	yes	yes	yes	yes	no, 594 underwent biopsy	yes			
Siddiqui et al. [10]	consecutive	yes	yes	yes	yes	yes	yes			
Lai et al. [11]	consecutive	yes	yes	yes	yes	No, 171 who had LSM ≥ 8, 71 underwent biopsy	yes	low	low	high
Chan et al. 2014 [12]	consecutive	yes	yes	yes	yes	yes	yes	low	low	high
Chan et al. 2017 [13]	consecutive	yes	yes	yes	yes	yes	yes	low	low	high
Jung et al. [14]	consecutive	yes	yes	yes	yes	yes	yes	low	low	low
Kwok et al. [15]	consecutive	yes	yes	yes	yes	no, only patients with advanced fibrosis or cirrhosis on VCTE	yes	low	low	high
Sasso et al. [16]	consecutive	yes	yes	yes	yes	yes	yes	low	low	low
Shen et al. [17]	consecutive	yes	yes	yes	yes	yes	yes	low	low	low
Karlas et al. [18]	consecutive	yes	yes	yes	yes	yes	yes	low	high	low
Chon et al. [19]	consecutive	yes	yes	yes	yes	yes	yes	low	low	low
Masaki [20]	consecutive	yes	yes	yes	yes	yes	yes	low	low	low
Kumar et al. [21]	consecutive	yes	yes	yes	yes	yes	yes	low	low	low
Friedrich-Rust et al. [22]	consecutive	yes	yes	yes	yes	yes	yes	low	low	low
Caussy et al. [23]	consecutive	yes	yes	yes	yes	yes	yes	low	low	low
Wong et al. [24]	consecutive	yes	yes	yes	yes	yes	yes	low	low	low
Tapper et al. [25]	consecutive	yes	yes	yes	yes	yes	yes	low	low	low
Bertrot et al. [26]	consecutive	yes	yes	yes	yes	yes	yes	low	low	low
Lee et al. [27]	consecutive	yes	yes	yes	yes	yes	yes	low	low	low
Liu et al. [28]	consecutive	yes	yes	yes	yes	yes	yes	low	low	low

**Table 2:** Characteristics of Studies Included in the Meta-Analysis.

Study	N	Type of Study	Location	% Male	Mean/ Median Age	BMI	% Completed Test	% with Diabetes	% with Hyper-Tension	% with Dyslipidemia	Type of Probe	Why People Refused
Boursier et al. [9]	1057	multicenter cohort	France, Sweden, Spain	62	55		76.8	37	44	27	M and XL	No show
Siddiqui et al. [10]	393	prospective	USA	32	51	34	not reported	44		57		
Lai et al. [11]	557	prospective cross-sectional	Malaysia	40.6	61	28.2	not reported	100	36.4	52.4	M and XL	NA
Chan et al. 2014 [12]	101	prospective cohort	Malaysia	51.5	50.3	29.4	not reported	52.5	88.1	95	M only	
Chan et al. 2017 [13]	57	prospective cross-sectional	Malaysia, Hong Kong	49	50.1	30.2	not reported				M	
Jung et al. [14]	161	prospective	Korea	63.4	49	24.4	not reported	17.4			M	
Kwok et al. [15]	1918	prospective cohort study	Hong Kong	54.3	61	26.6	90.4	100	69.9	67.6	M and XL	
Sasso et al. [16]	112	prospective study	France	54	53.8	25.8	not reported	24	37		M	
Shen et al. [17]	152	multicenter prospective	China	69.3	35	26	not reported				M	
Karlas et al. [18]	50	prospective cohort study	Germany	25	54.7 +/− 9.1	33.0 +/− 4.9	not reported	50	67		M	
Chon et al. [19]	135	prospective study	Korea	64	51	24.4	not reported				M	
Masaki et al. [20]	150		Japan	61.3	55	24.4	not reported				M	
Kumar et al. [21]	317		India	73	37	25.1 +/− 2.0	not reported				M	
Friedrich-Rust et al. [22]	57		Germany	52.6	45 +/− 14	28 +/− 5.5	not reported				M and XL	
Caussy et al. [23]	119	cross-section prospective	California, San Diego (UCSD)	41.2	52.4	29.9	95				X and M	No show
Wong et al. [24]	246	prospective cohort	France, China	54.9	51 +/− 11	28 +/− 4.5	not reported	36.2	40.2		M	
Tapper et al. [25]	164	prospective cohort	USA				91.4 (3 m) 53 (6 m)				M	
Bertrot et al. [26]	271	retrospective cohort	Australia	40	52 +/− 12	38 +/− 8	not reported	49	45	27	M and XL	
Lee et al. [27]	251	multi-center retrospective cohort	Korea	52.6	44	28.64	87.2	46.6	31.1		M and XL	
Liu et al. [28]	101	prospective cohort study	China	16.8	38.9 +/− 10.8	41.1 +/− 5.6	94.6 (1 y) 100 (2 y) 94.6 (3 y) 91.9 (4 y) 64.9 (5 y)	48.6	51.4	43.2	M and XL	

**Table 3:** Summary Diagnostic Performance of Point-of-Care Fibroscan for the Detection of Fibrosis Stages and CAP.

	Studies, (Patients; *n*)	AUC	Sensitivity (95%CI)	Specificity (95%CI)	Negative Likelihood Ratio (95% CI)	Positive Likelihood Ratio (95% CI)	Diagnostic Odds Ratio (95% CI)
F ≥ 1	4 (210)	0.74	69.5% (0.49–0.84)	70.6% (0.29–0.93)	0.47 (0.31–0.71)	2.93 (1.13–8.23)	6.62 (1.64–18.30)
F ≥ 2	8 (650)	0.69	81.7% (0.62–0.92)	64.6% (0.56–0.73)	0.30 (0.12–0.58)	2.30 (1.67–3.02)	9.28 (2.96–22.30)
F ≥ 3	10 (619)	0.84	88.1% (0.78–0.94)	63.8% (0.49–0.77)	0.20 (0.10–0.36)	2.50 (1.68–3.77)	14.60 (5.02–33.50)
F ≥ 4	9 (543)	0.65	87.5% (0.78–0.93)	62.6% (0.55–0.70)	0.21 (0.11–0.36)	2.34 (1.86–2.92)	12.70 (5.36–25.70)
CAP < 33%	10 (510)	0.85	84.3% (0.81–0.94)	70.3% (0.55–0.82)	0.23 (0.17–0.31)	2.94 (1.88–4.65)	13.40 (6.59–24.40)
CAP 34–66%	12 (309)	0.75	76.5% (0.68–0.83)	54.0% (0.40–0.68)	0.45 (0.29–0.68)	1.70 (1.23–2.40)	4.11 (1.83–8.00)
CAP ≥ 67%	12 (518)	0.80	80.6% (0.75–0.85)	57.9% (0.45–0.70)	0.35 (0.23–0.51)	1.95 (1.42–2.71)	6.07 (2.85–11.40)

**Table 4:** Probe Type (M, M, and XL or Not Specified Studies) as a Covariate of the Diagnostic Performance.

	M	M and XL	Not Specified	Chi-Square	*p*-Value
Any fibrosis (F ≥ 1)	11	34	60	12.13	0.0164
Significant fibrosis (F ≥ 2)	29	362	47	10.536	0.0323
Advanced fibrosis (F ≥ 3)	27	354	44	8.2596	0.0825
Cirrhosis (F = 4)	54	297	24	9.0037	0.0610
CAP < 33%	209	22	94	11.0670	0.0258
CAP 34–66%	55	71	40	7.3715	0.1175
CAP >= 67%	169	71	63	9.8008	0.0439

**Table 5:** BMI as a Covariate of the Diagnostic Performance.

	Chi-Square	*p*-Value
Fibrosis	0.3842	0.8252
Steatosis	7.292	0.0261

**Table 6:** Male Percentage as a Covariate of the Diagnostic Performance.

	Chi-Square	*p*-Value
Fibrosis	9.0215	0.0110
Steatosis	10.9400	0.0042

**Table 7:** Fibrosis or Steatosis Level as a Covariate of the Diagnostic Performance.

	Chi-Square	*p*-Value
Figure 7	7.0286	0.3182
Steatosis	3.032	0.5525

## Data Availability

No new data were created or analyzed in this study.
